# ES-ImageNet: A Million Event-Stream Classification Dataset for Spiking Neural Networks

**DOI:** 10.3389/fnins.2021.726582

**Published:** 2021-11-25

**Authors:** Yihan Lin, Wei Ding, Shaohua Qiang, Lei Deng, Guoqi Li

**Affiliations:** Department of Precision Instrument, Center for Brain Inspired Computing Research, Tsinghua University, Beijing, China

**Keywords:** data set, spiking neural network, dynamic vision sensor, brain inspire computation, leaky integrate and fire

## Abstract

With event-driven algorithms, especially spiking neural networks (SNNs), achieving continuous improvement in neuromorphic vision processing, a more challenging event-stream dataset is urgently needed. However, it is well-known that creating an ES-dataset is a time-consuming and costly task with neuromorphic cameras like dynamic vision sensors (DVS). In this work, we propose a fast and effective algorithm termed Omnidirectional Discrete Gradient (ODG) to convert the popular computer vision dataset ILSVRC2012 into its event-stream (ES) version, generating about 1,300,000 frame-based images into ES-samples in 1,000 categories. In this way, we propose an ES-dataset called ES-ImageNet, which is dozens of times larger than other neuromorphic classification datasets at present and completely generated by the software. The ODG algorithm implements image motion to generate local value changes with discrete gradient information in different directions, providing a low-cost and high-speed method for converting frame-based images into event streams, along with Edge-Integral to reconstruct the high-quality images from event streams. Furthermore, we analyze the statistics of ES-ImageNet in multiple ways, and a performance benchmark of the dataset is also provided using both famous deep neural network algorithms and spiking neural network algorithms. We believe that this work shall provide a new large-scale benchmark dataset for SNNs and neuromorphic vision.

## Introduction

In recent years, spiking neural networks (SNNs) have attracted extensive attention in the fields of computational neuroscience, artificial intelligence, and brain-inspired computing (Pei et al., 2019; Roy et al., 2019). Known as the third generation of neural networks (Maass, 1997), SNNs have the ability to process spatiotemporal information and own stronger biological interpretability than artificial neural networks (ANNs, or deep neural networks). They have been applied in a number of tasks, such as pattern recognition (Schrauwen et al., 2008; Rouat et al., 2013; Zhang et al., 2015), high-speed object tracking (Yang et al., 2019), and optical flow estimation (Paredes-Vallés et al., 2019) with the help of neuromorphic hardware such as TrueNorth (Akopyan et al., 2015), Loihi (Davies et al., 2018), DaDianNao (Tao et al., 2016), and Tianjic (Pei et al., 2019). In recent years, the continuous expansion of datasets in image classification (LeCun et al., 1998; Deng et al., 2009; Krizhevsky and Hinton, 2009), natural language processing (Nguyen et al., 2016; Rajpurkar et al., 2016), and other fields has been challenging the ability of AI and promoting the development of AI. The researchers hope that AI can surpass humans in the corresponding tasks. However, for the SNNs, the research is still in the rising stage with obstacles gradually appearing, where the lack of suitable datasets is one of the biggest ones. We now have *N-MNIST* (Orchard et al., 2015), *N-Caltech101* (Orchard et al., 2015), *DVS-Gesture* (Amir et al., 2017), *CIFAR10-DVS* (Li et al., 2017), and other neuromorphic datasets (or event-stream datasets, ES-datasets), but those existing datasets designed for SNNs are relatively small in scale. As more algorithms are proposed, the scale of SNNs is growing larger. Therefore, the existing datasets have found it difficult to meet the demand for training and validation of SNNs.

**Figure 1:**
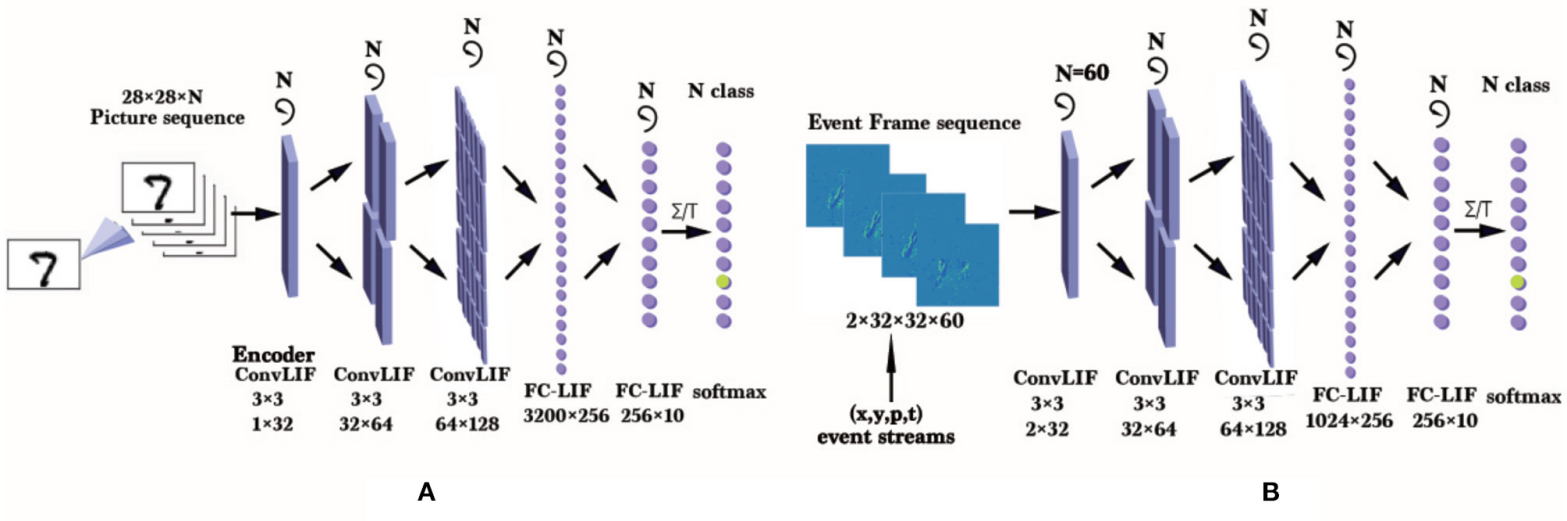
**(A)** An approach for training the LIF-SNN (Dayan and Abbott, 2001) on an ANN-oriented dataset. Here, the SNN uses rate coding and an ANN-like structure, so it can be trained using frame data naturally. **(B)** Training an LIF-SNN with GPUs on a DVS-dataset (ES-dataset recorded by DVS). Here we need to accumulate events within a small period as an event frame and get an event frame sequence with *N* frames for training. On the neuromorphic processor, these asynchronous event data can be processed more efficiently.

A compromised solution towards this problem is to train SNNs on the large-scale traditional static datasets directly. Taking image classification for instance, the common method is copying an image multiple times to form an image sequence, and then the sequence is fed into the spike encoding layer of an SNN, as Figure 1A shows. However, there is an obvious shortcoming that the data redundancy makes the training cost increase many times without any effective information being added. For comparison, the way to train an SNN on an ES-dataset is also shown in Figure 1B. Compared to the common method, it is more natural for SNNs to process such sparse and temporal data by making full use of temporal characteristics. So the datasets inspired by the neuromorphic visual sensor imaging mechanism are still considered to be the most suitable datasets for SNNs.

Since SNNs have benefited from neuromorphic data, efforts are also devoted to recycling the existing RGB-cameras datasets to generate neuromorphic datasets. Mainly there are two different methods for this task. One is to use dynamic vision sensor (DVS) cameras to record a video generated from raw data with an LCD screen (Orchard et al., 2015; Li et al., 2017). This method is time-consuming and costly, which is impossible for manufacturing a large-scale dataset. The other one is to generate the event data using software to simulate the principle of DVS cameras (Bi and Andreopoulos, 2017; Gehrig et al., 2020). This kind of method is more suitable for generating large-scale event-based datasets. However, the data redundancy caused by the existing converting algorithms increases the volume of the datasets. In this work, we optimize the existing algorithms of the second method to obtain the dataset with less redundancy.

In this way, an ES-dataset converted from the famous image classification dataset *ILSVRC2012* (Russakovsky et al., 2015) is generated, which is named *event-stream ImageNet* or *ES-ImageNet*. In *ES-ImageNet*, there are about 1.3 M samples converted from *ILSVRC2012* in 1,000 different categories. *ES-ImageNet* is now the largest ES-dataset for object classification at present. We have sorted out the information of representative existing ES-datasets and compared them with *ES-ImageNet*, the results are summarized in Table 1. Having more categories and samples also probably makes it the most challenging classification ES-dataset, providing space for continuous improvement of event-driven algorithms.

**Table 1:** Comparison between existing ES-datasets and ES-ImageNet.

**Name**	**Generating speed^a^**	**Resolution**	**# of samples**	**# number**	**# Type**
POKER-DVS (Prez-Carrasco et al., 2013)	–	32× 32	131	4	Classify
N-MNIST (Orchard et al., 2015)	300 ms/sample	28× 28	60, 000 training + 10, 000 test	10	Classify
DVS-Caltech101 (Orchard et al., 2015)	300 ms/sample	302× 245 on average	8709	100	Classify
DVS-UCF-50 (Hu et al., 2016)	6,800 ms/sample	240× 180	6,676	50	Classify
DVS-Caltech-256 (Hu et al., 2016)	1,010 ms/sample	240× 180	30,607	257	Classify
DVS-VOT-2015 (Hu et al., 2016)	30 FPS, 20.70 s/sample	240× 180	67	–	Track
DVS-CIFAR10 (Li et al., 2017)	300 ms/sample	512× 512	10,000	10	Classify
DVS-Gesture (Amir et al., 2017)	6 s/sample	128× 128	1,342	11	Classify
Pred-18 (Moeys et al., 2018)	15 FPS	240× 180	1.25 h (67.5k frames)	2	Detect
Action Recognition (Miao et al., 2019)	5 s/sample	346× 260	450	10	Classify
1Mpx Detection Dataset (de Tournemire et al., 2020)	60 s/sample	304× 240	14.65 h, 255,781 objects	2	Detect
SL-ANIMALS-DVS (Vasudevan et al., 2020)	–	128× 128	1,102	10	Classify
DVS-Gait-Day/Night (Wang et al., 2021)	3–4 s/sample	128× 128	4,000	20	Classify
N-ROD (Cannici et al., 2021)	6.6 s/sample	256× 256	41,877	51	Classify
ES-ImageNet	29.47 ms/sample^b^	224× 224^c^	1,257,035 training + 49,881 test	1000	Classify

a *The average time taken for generating each sample or average recording time (for directly recorded)*.

b *Threshold = 0.18*.

c *The events are generated in a range of 256× 256 pixels. But only those in the central 224× 224 pixels are meaningful, while others are noise-generated by the image edge's motion*.

A good conversion algorithm is expected to generate a dataset that is smaller than the source. If it is not required to imitate the characteristics of DVS, the optimal binary-coding conversion is able to encode the original information with the same size of data. So when the conversion algorithm generates a larger dataset than the original one, there must be data redundancy. In order to simulate the DVS cameras, we can allow a little redundancy. However, most of the existing conversion methods generate a much larger dataset [for example, N-MNIST (Orchard et al., 2015) and Flash-MNIST's storage volume is tens of GB, where the original MNIST is no larger than 100 MB]. This is far from the original intention of DVS sparsity, and it is not conducive to high-speed efficient processing and large-scale applications. So a simple bio-inspired algorithm called *Omnidirectional Discrete Gradient* (ODG) is applied. This algorithm captures the sequential features of images and then places them on the time axis with timestamps to generate event streams. It reduces the information redundancy, which brings higher generation speed and less data redundancy than the existing conversion algorithms. It can be regarded as the streamlining of random saccades for deep learning use, where the latter is a common bio-inspired generation method.

To guarantee a suitable sparsity of data and the amount of information, we also conduct preparatory experiments to control the event rates and the amount of information of the generated samples. Further analysis about the computation cost of different algorithms is conducted, which confirms that the dataset is an SNN-friendly dataset.

The main contributions of this work are 3-fold.

(i) We introduce a new large-scale ES-dataset named *ES-ImageNet*, which is aimed at examining SNNs' ability to extract features from sparse event data and boosting research on neuromorphic vision. This work shall provide a new large-scale benchmark dataset for SNNs and neuromorphic vision tasks.

(ii) A new algorithm called ODG is proposed for converting data to its event stream version. We consider the guiding ideology behind it to be a paradigm for conversion from static data to ES-data, which avoids data redundancy.

(iii) Several ways for analyzing the dataset are provided, including information loss analysis using 2D information entropy (2D-Entropy) and the visual perception of the reconstructed pictures. Also two preparatory experiments are designed for designing the algorithm, which may provide inspiration for further improvement.

### Related Work

#### ES-Datasets Collected Directly From the Real Scenarios

DVS cameras can generate unlabeled ES data directly (Brandli et al., 2014). The ES data are often organized as a quad (*x, y, t, p*), where (*x, y*) are the topological coordinates of the pixel, *t* is the time of spike generation, and *p* is the polarity of the spike. Such datasets are easy to generate and close to practical application scenarios, like datasets that can be used for tracking and detection (Bardow et al., 2016; Moeys et al., 2018; de Tournemire et al., 2020), datasets for 3D scene reconstruction (Carneiro et al., 2013; Kim et al., 2016), neural morphology datasets for optical flow estimation (Benosman et al., 2013; Bardow et al., 2016), and datasets for gesture recognition (Amir et al., 2017). Due to the high sampling rate and authenticity, these kinds of datasets are of great help to the development of applications in high-speed scenes. But because of the huge workload of making real scenario recording datasets, their sizes are often small, which is difficult to meet the demand of examining deep SNNs algorithms.

#### Transformed ES-Datasets With Help of Neuromorphic Sensors

These datasets are mainly generated by the labeled static image datasets through neuromorphic sensors. Different from the first ones, these kinds of datasets are mainly obtained from the datasets which have been widely studied and used for traditional ANN tasks, such as *N-MNIST* (Orchard et al., 2015), *DVS-UCF-50, DVS-Caltech-256* (Hu et al., 2016), and *CIFAR10-DVS* (Li et al., 2017). In order to make such datasets, one way is to use a screen to display a static picture, then face the DVS camera to the screen and move the camera along the designed trajectory to generate events. Because of the similarity between the transformed dataset and the original one, this kind of dataset can be used and evaluated easily. Therefore, they are also the most widely used datasets in SNN research. However, during the recording process, noise is introduced, especially caused by the flashing LCD screen.

#### Completely Software-Generated ES-Datasets Without Neuromorphic Sensors

The algorithms are used to simulate the characteristics of DVS cameras with labeled data here. The dynamic sensors can capture the dynamic information from the video streams or picture sequences, while this process can also be completed by specific algorithms (Bi and Andreopoulos, 2017; Yang et al., 2019; Gehrig et al., 2020). These methods can avoid a large number of experiments needed for collecting data. However, the existing algorithms used for converting static data to event data always extract information with too much redundancy that is brought by the randomness or repetitiveness of the generation algorithms.

## Materials and Methods

In this section, we will introduce a method to generate the *ES-ImageNet* with a corresponding reconstruction method, including the color space conversion, ODG processing, hyper-parameters choosing, and sparse storage.

### Color Space Conversion

Traditional ES-datasets utilize DVS cameras to record the changes of intensity asynchronously in the ES format, which encode per-pixel brightness changes. In RGB (red-green-blue) color models, a pixel's color can be described as a triplet (*red, green, blue*) or (*R, G, B*), which does not indicate brightness information directly. When using a HSV (hue-saturation-value) color model, it is described as (*hue, saturation, value*) or (*H, S, V*). Generally, the images in the *ILSVRC2012* dataset are stored in the RGB color space, therefore images need to be converted to the HSV color space, as shown in


(1) $$H=\{\begin{array}{rr} 0^{{}^{\circ}} & if\;M=m\\ 60^{\times }\frac{G-B}{M-m}+0^{{}^{\circ}} & if\;M=R\;and\;G>B\\ \;60^{{}^{\circ}}\times \frac{G-B}{M-m}+360^{{}^{\circ}} & if\;M=R\;and\;G\leq B\\ 60^{{}^{\circ}}\times \frac{B-R}{M-m}+120^{{}^{\circ}} & if\;M=G\\ 60^{{}^{\circ}}\times \frac{R-G}{M-m}+240^{{}^{\circ}} & if\;M=B \end{array}$$

(2) $$S=\{\begin{array}{rr} 0 & if\;\max=0\\ \frac{M-m}{M}=1-\frac{m}{M} & otherwise \end{array}$$

(3) V=M,

where *M* = max{*R, G, B*} and *m* = min{*R, G, B*}. In this algorithm, we use *V* as a reference of light intensity. In the HSV hex-cone model, the value indicates the brightness of the color. And for the light source, the value is also related to the brightness of the illuminant, so it can be used as a reference for light intensity.

### Event Generator

To stimulate the intensity changes, we use ODG here. Based on the truth that animals like toads or frogs can only respond to moving objects (Ewert, 1974), we believe that we can obtain the necessary information for object recognition by imitating the frog retinal nerves, specifically, ganglion cells that generate features. Three important kinds of ganglion cells act as edge detectors, convex edge detectors, and contrast detectors, generating sparse local edge information. This inspires the main idea of ODG, which is artificially changing the light intensity and detecting the necessary local edge information in multiple directions.

**Figure 2:**
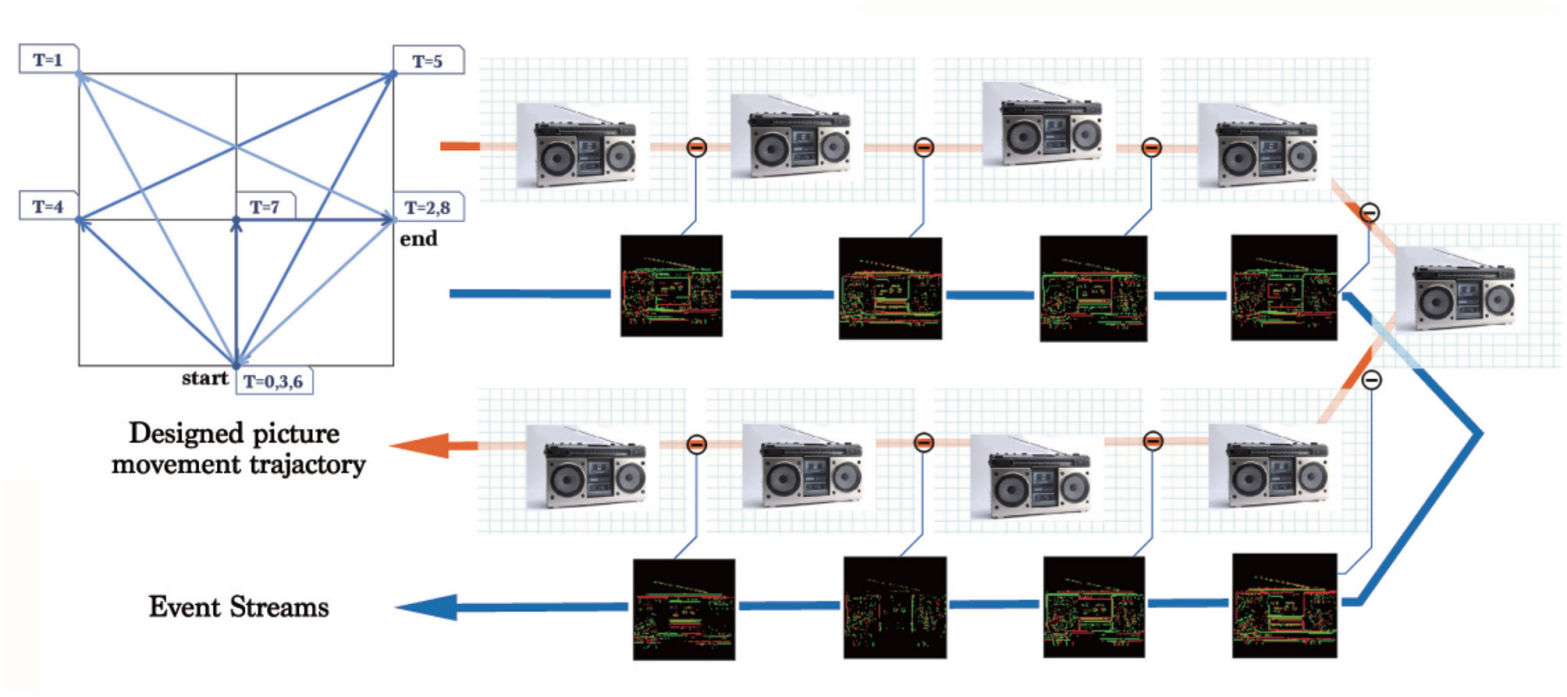
The moving trajectory of images used to generate the events. The numbers in the small blue squares is the timestamp when an image reaches the corresponding position. The pipeline shows the complete process of generating an event stream.

Different from the widely used random saccades generation (Hu et al., 2016), we only choose necessary directions in a fixed order and the necessary number of frames to minimize data redundancy, we will explain it later. This algorithm generates an event stream for each picture in *ILSVRC2012* with a specific moving path shown in Figure 2, and the algorithm is summarized in Algorithm 1. The trigger condition of the events is described in


(4) $$\;\;\;p(x,y,t)=\{\begin{array}{ll} -1 & if\;\;V(x,y,t)-V(x,y,t-1)<-Thresh\\ \;1 & if\;V(x,y,t)-V(x,y,t-1)>Thresh, \end{array}$$

where *p*(*x, y, t*) denotes the polarity of the event at (*x, y, t*), *V* is the value of pixel, and *Thresh* is the difference threshold. This algorithm only involves linear operations with time complexity of $O\left(W^{2}T\right)$, where *W* denotes the width of the image and *T* is the length of time. ES-ImageNet is generated without randomness so that users can reconstruct the original information using the path information and design data augmentation freely.

**Algorithm 1:** ODG event generator.

**Require:** *Image*
**Ensure:** *Stream*
xTrace = [1,0,2,1,0,2,1,1,2], yTrace = [0,2,1,0,1,2,0,1,1], Thresh = 0.18, T = 8
**function** GENERATOR(Image)
W = = Image.size[0], H = Image.size[1]
Image = zeroPadding(upSampling(Image, (254, 254)), 2)
V = RGB2HSV(Image).V
**for** t = 0 → T **do**
x = xTrace[t], y = yTrace[t]
newImage = V[x : x+W, y : y+H]
**if** t > 0 **then**
oldX = xTrace[t-1], oldY = yTrace[t-1]
ImgDiff = newImage - lastImage
posEvent = ImgDiff(ImgDiff ≥ Thresh), negEvent = ImgDiff(ImgDiff ≤ -Thresh)
**for** i = 0 → len(posEvent) **do**
Ex = posEvent[0], Ey = posEvent[1]
**if** (Ex, Ey) is in valid range **then**
posStream.append((Ex, Ey,t))
**end if**
**end for**
**for** i = 0 → len(negEvent) **do**
Ex = negEvent[0], Ey = negEvent[1]
**if** (Ex,Ey) is in valid range **then**
negStream.append((Ex,Ey,t))
**end if**
**end for**
**end if**
lastImage = newImage
**end for**
**end function**

In Algorithm 1, there are four hyper-parameters to be selected: a sequence of the x coordinate (*xTrace*), a sequence of the y coordinate (*yTrace*), the difference threshold (*Thresh*) in Equation (4), and the number of time steps (*T*). We designed two preparatory experiments to determine these hyper-parameters.

### Select the Hyper-Parameters

#### Trajectory

**Figure 3:**
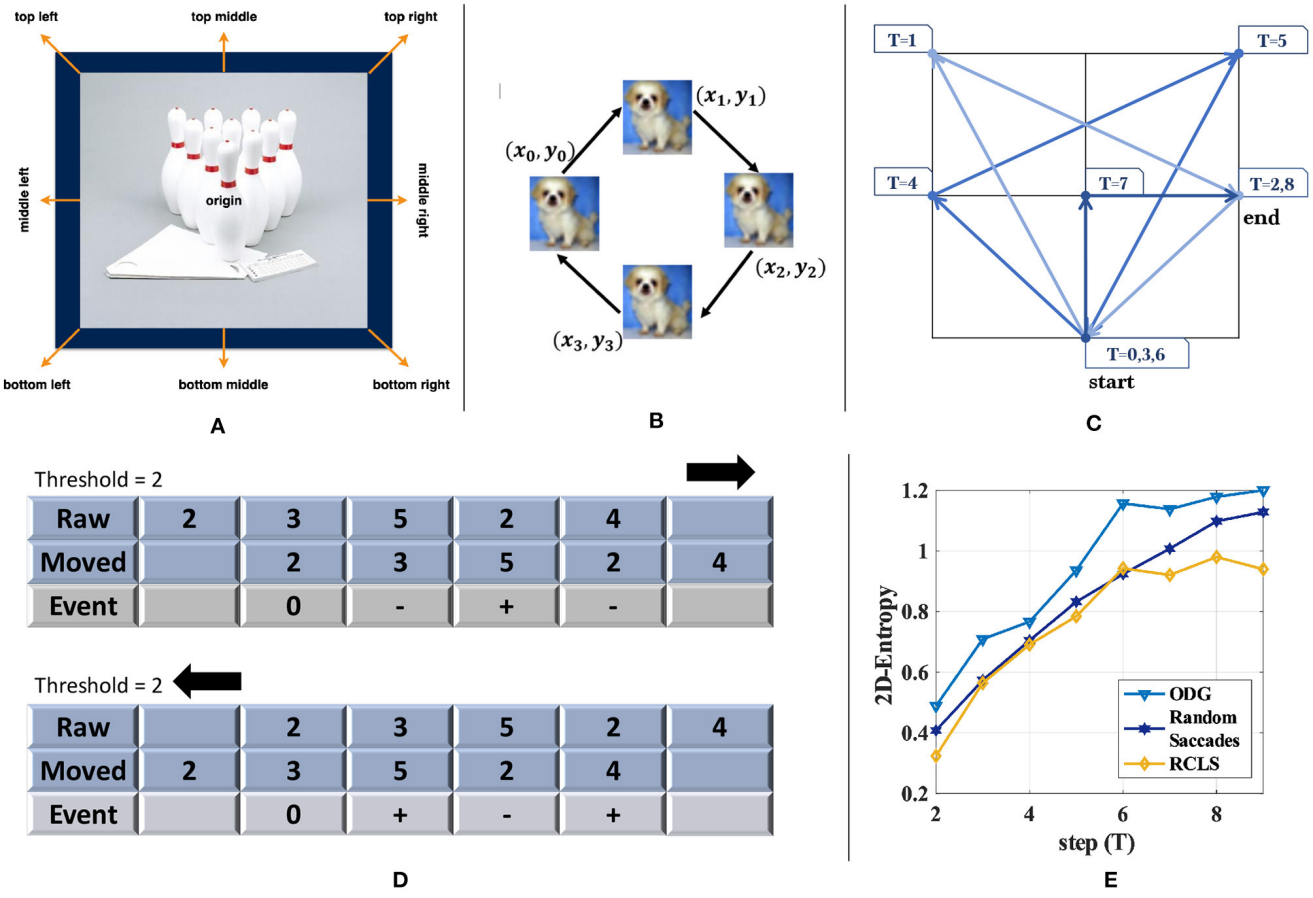
Comparison of three different kinds of image motion. **(A)** The candidate moving directions used in random saccades generation of DVS-Caltech-256 and DVS-UCF-50 (Hu et al., 2016). **(B)** The path used in RCLS of DVS-CIFAR-10 (Li et al., 2017). **(C)** Trajectory used in ES-ImageNet. **(D)** Illustration to explain why opposite directions in the generation path would only generate opposite events. **(E)** 2D-Entropy comparison among the three generating paths with different steps (*T*). ODG is superior to the other two methods in the sense of 2D-Entropy based on the reconstructed gray images.

The choice of the path is important, which includes designing *xTrace* and *yTrace* along with choosing *T*. Most of the existing conversion methods choose fast random saccades or repeated fixed paths. The former selects eight directions for simulating fast eye movement (random saccades), while the latter uses only four directions [repeated closed-loop smooth (RCLS)], as shown in Figures 3A–C.

To verify the information obtained by these different methods, we evaluate it by comparing their 2D information entropy (2D-Entropy), which is positively correlated with the amount of information in an image. The average neighborhood gray value of the image is selected to represent the spatial characteristics, and a 3 × 3 field is commonly used. The feature pair (*i,j*) is used to calculate 2D-entropy, where *i* is the gray value of the pixel, and *j* is the rounding down of the mean neighborhood gray value. The 2D-Entropy of the gray image is defined as Equation (5), where *p*_(*i,j*)_ denotes the frequency of the feature pair (*i, j*) and *g* is the gray level.


(5) $$H=\sum_{i=0}^{g}\sum_{j=0}^{g}-p_{\left(i,j\right)}log_{2}(p_{\left(i,j\right)}).$$

Because these algorithms use the frame difference for event generation, and the adjacent frames are actually the same image, the movement with the opposite direction would always generate events with opposite polarity. Therefore, a new step with an opposite direction to the existing movement does not add more effective information into the sample, and that is how the existing algorithms can be optimized. As shown in Figure 3D, the number in the cell denotes the pixel value. A row of pixels move left or right, and calculating the difference under the same threshold would only obtain a series of events with exactly the opposite polarity.

Based on this observation, we avoid the repeated or the opposite path design in the *ODG*. Furthermore, to quantitatively illustrate the benefits, we randomly select 100 images from ImageNet-1K, extract events in different *T* with the three different methods, and then reconstruct them into gray images to calculate 2D-Entropy. In this way, we get Figure 3E, and the higher curve of ODG may support our modification.

Through analyzing the information (2D-Entropy) curves calculated for each method over several time steps in Figure 3E, we find that the 2D-Entropy increases slowly after *T* ≥ 6, but the size of the dataset would still increases linearly with *T* getting larger. In order to make a balance between the temporal characteristics, the amount of information and the size of dataset, we set *T* = 8.

#### Threshold

**Figure 4:**
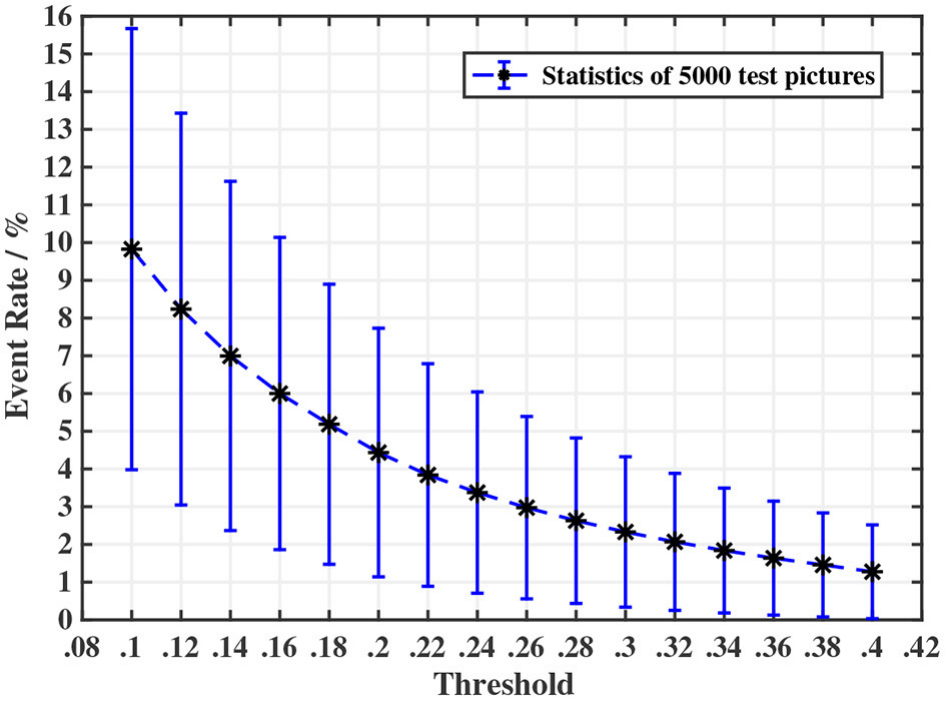
A preparatory experiment for determining the threshold in ODG. We observed that the event rate shows a trend of an exponential decrease in the threshold. In consideration of the event rate of generated samples and to avoid generating too many invalid samples that have an extremely low event rate, we choose *threshold* = 0.18.

An important indicator for the ES-dataset is event rate, which is defined as the proportion of pixels that have triggered an event. The most influential parameter for event rate is the threshold *Thresh* (when the motion path is fixed). Because of the high correlation of brightness between adjacent pixels, it is hard to estimate the distribution of the difference between adjacent pixels using the static method, so a preparatory experiment is needed. We randomly select 5 pictures from each category and get 5,000 pictures. The threshold in the experiment varies from 0.1 to 0.4. The results are shown in Figure 4. After many tests, we choose 0.18 as the threshold value, for an estimated event rate of 5.186%, with the event rate of most samples being in the range of 1 to 10%. This result will be verified on the whole dataset. It should be noted that many events may be generated by the movement of the edge of the image, and they have been wiped out.

In addition, the original images' longest sides are normalized to 255 by the nearest-interpolation algorithm. Nearest interpolation is mapping from the normalized coordinates after zooming into the integer grid coordinates. The generated event stream version training set is 99 GB and test set is 4.2 GB, which are stored in the quad format (*x, y, t, p*). If converted to a frame version like a short video, the size of the whole dataset can be further reduced to 37.4 GB without information loss. For ease of use, we store all of these tensors as a file in the .npz format, using the scientific computing package "Numpy" of Python. The event-frame format version is more suitable for deep learning, and we will also provide this version, while the quad format version is the classical ES-dataset.

### Data Analysis

#### Event Rate

To examine the quality of the data, we calculate the event rates of the whole generated dataset and summarize them in Table 2. It can be seen that the pixels which trigger the events are only about one-twentieth of all pixels. And from this point of view, the prediction obtained from the preparatory experiment is accurate. Since our events are generated from the images processed by nearest-neighbor interpolation, our event rate statistics are also calculated in this range. When we use the training data, we often place the positive events and negative events in different channels and organize them in the unified 2 × 224 × 224 (*C* × *W* × *H*) frame format. Therefore, we re-calculate the event rate during the training process in Table 2, which is lower than that of the generating process and is more meaningful for the training process.

**Table 2:** Event rate of ES-ImageNet.

**Generating process**	**Training set**		**Testing set**	
	**Mean**	**σ**	**Mean**	**σ**
Events	5.215%	3.776%	5.385%	3.837%
ON	5.211%	3.777%	5.385%	3.838%
OFF	5.22%	3.78%	5.38%	3.84%
**Event-frame format**	**Training set**		**Testing set**	
	**Mean**	σ	**Mean**	**σ**
Events	4.461%	3.560%	5.231%	3.770%
ON	4.458%	3.570%	5.229%	3.770%
OFF	4.460%	3.560%	5.230%	3.770%

**Figure 5:**
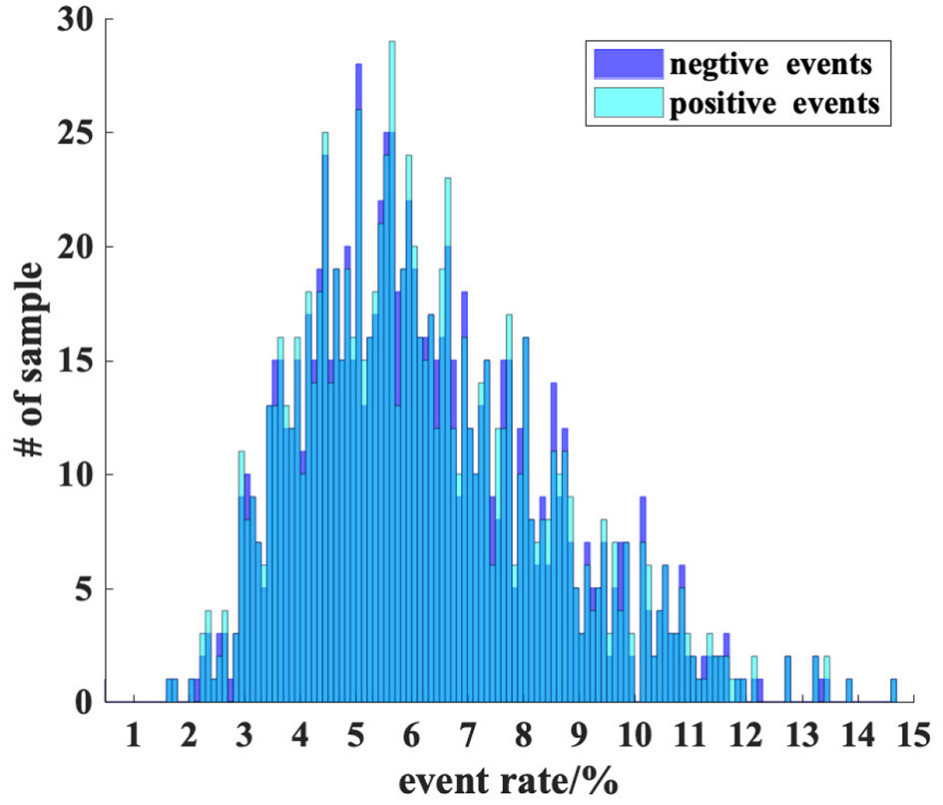
A detailed inspection about fire rate. Most samples have a 5 to 6% event rate, and this figure shows a significant skew distribution. A sample with a 5% event rate is also at a relatively sparse level when recorded by event cameras.

Furthermore, we calculate the distribution histogram of positive and negative events and show it in Figure 5. The results in the figure show that the distribution of positive and negative events is very close, which may be because most of the entities in the original images are represented as closed graphics.

#### Visualization

**Figure 6:**
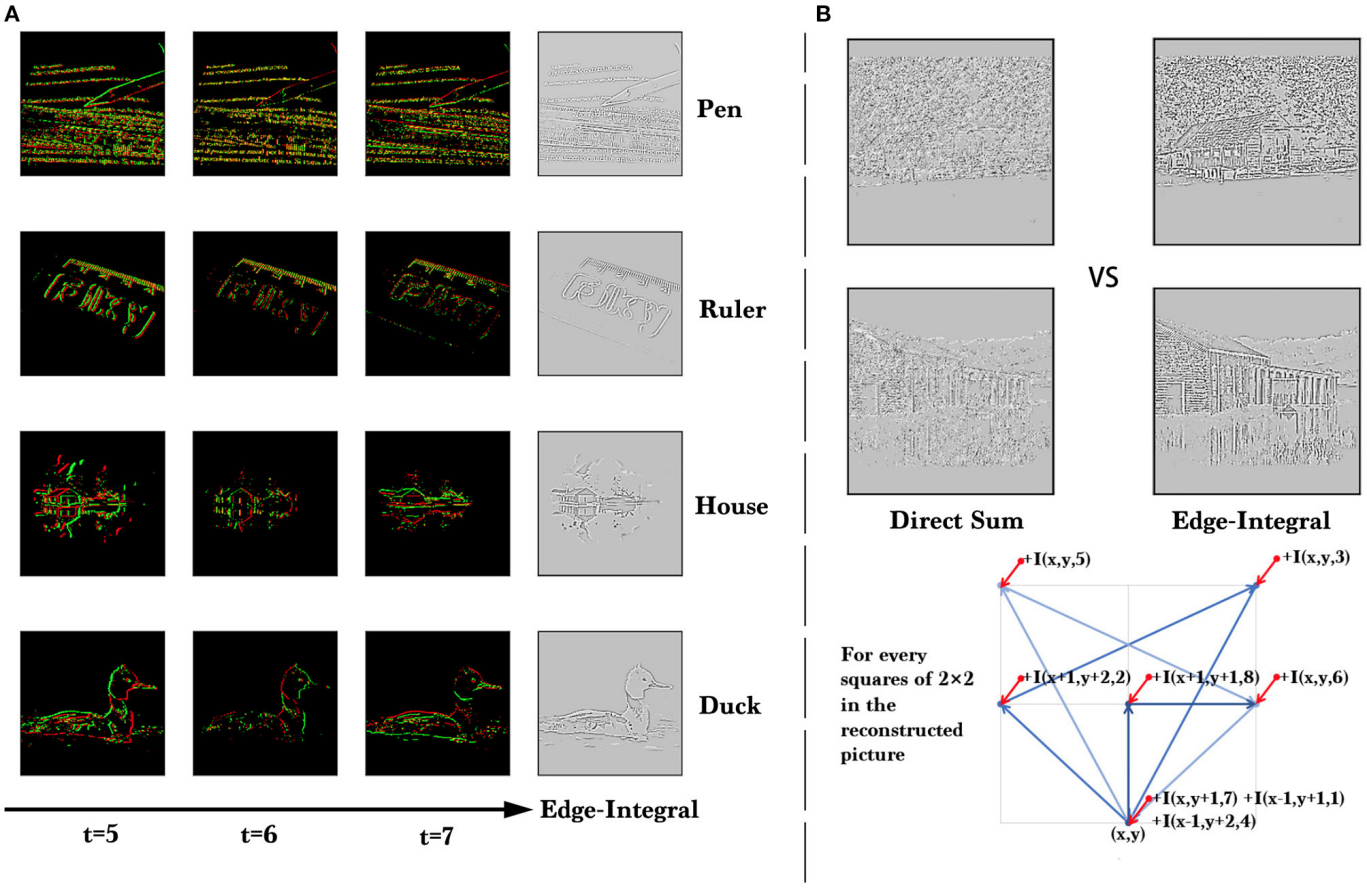
**(A)** The visualization of ES-ImageNet. We show a few samples reconstructed from event streams and the event frames at the last three time steps for each sample. These examples are from four different categories and can be clearly identified. **(B)** Quality comparison of the reconstruction results of direct summation and Edge-Integral. Here *I*(*x, y, t*) means the intensity in (x,y) in the event frame at *time* = *t*.

To show the quality of the data intuitively, we reconstruct the original pictures from event streams. Firstly we accumulate the events into frames, and we obtain eight (*T* = 8) event frames. Different from the traditional DVS-dataset, our dataset is generated along a fixed path with multiple directions, so when we try to reconstruct the original pictures, we need to accumulate the difference frames (so called Edge-Integral used in Le Moigne and Tilton, 1995) along the opposite direction of the generating path. The results are shown in Figure 6, and the pseudo-code of Edge-Integral can be found in Algorithm 2. A visualization demo can be found in the Supplementary Materials.

**Algorithm 2:** Edge-Integral.

**Require:** *imageList*
**Ensure:** *grayImage*
**function** GENERATOR(Image)
Xtrace = [1,0,2,1,0,2,1,1,2]
Ytrace = [0,2,1,0,1,2,0,1,1]
imSize = size(imageList[0])
H = imSize[0], W= imSize[1]
SUM = zeros(H+4,W+4)
T = length(ImageList)
**for** *t* = 0 → *T* **do**
dx = Xtrace[j]
dy = Ytrace[j]
frame=imageList[t]
SUM[2-dx:2-dx+H,2-dy:2-dy+W] += frame[0]
SUM[2-dx:2-dx+H,2-dy:2-dy+W] -= frame[1]
**end for**
*gray*_*image* = SUM
**end function**

Analyzing the process of conversion, we know that there are three operations potentially causing the information loss. Firstly, only the information in the V channel of the HSV color space is used, and secondly, the gradient information obtained is also approximate, while thirdly, the information is stored in low-bit. According to the method in Figure 6, we are able to reconstruct the gray images, which can also be directly obtained from the original color images by the weighted sum of (*R, G, B*).

#### Information

To further analyze the loss of information during the conversion, we still turn to the 2D-Entropy of the gray images defined in Equation (5). We randomly collect 5000 RGB-images in *ILSVRC2012* (5 per class) and convert them into gray images with 256 gray levels, 17 gray levels, and 5 gray levels, respectively. And then we find the converted samples of those RGB-images in *ES-ImageNet* and reconstruct the corresponding gray images. Because the default is *T* = 8 in *ES-ImageNet*, and each pixel value could be 0, 1, or −1, the reconstructed samples will have a total of 17 gray levels (from 0 to 16).

**Figure 7:**
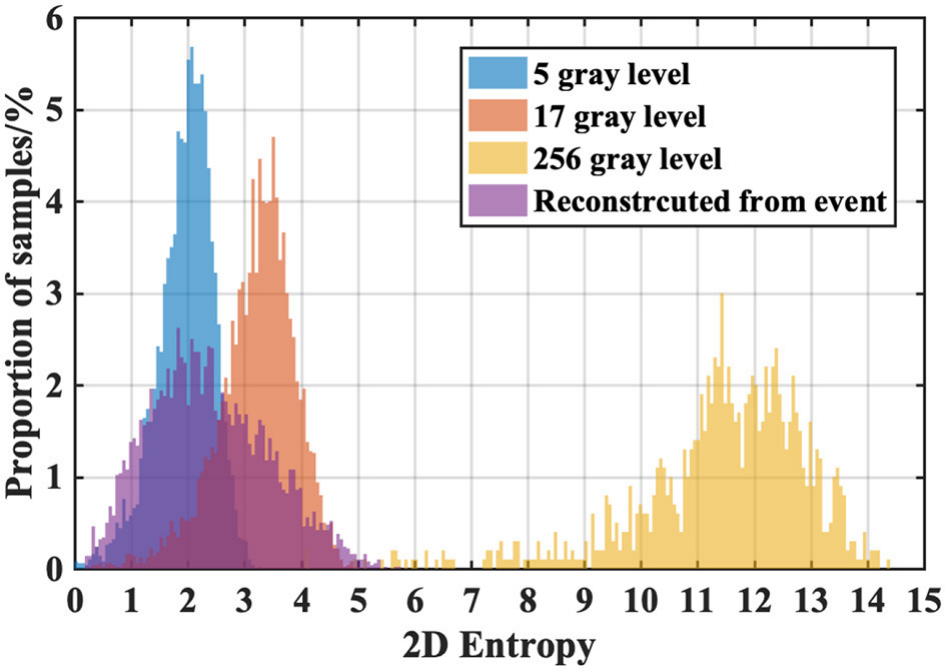
2D-Entropy histogram of the different compression levels of ILSVRC2012 sample groups and the reconstructed ES-ImageNet sample group. We compare the 2D-Entropy of four sample groups here. The reconstructed group indicates that the samples in ES-ImageNet potentially have effective information for object classification.

The ordinal meaning of 2D-Entropy can tell us what level the amount of information of *ES-ImageNet* is, which is no less than that with 5 gray-level compressed RGB-images and almost the same as that with 17 gray-level compressed RGB-images, as Figure 7 shows. It should be noted that the reconstruction process also causes information loss, so the original *ES-ImageNet* may have more efficient information than we speculate. Considering that the application of neural morphological data does not need many high-level features, we believe that the amount of information can make this dataset a nice validation tool of SNN.

## Results

### Training Experiments

Because the size of this dataset is very large, it is difficult to train a classical classifier (such as K-nearest neighbor) on it compared to other DVS-datasets (Li et al., 2017). Statistical learning methods such as support vector machine (SVM) do not perform well on large-scale datasets with many categories, and it might take days to train a vanilla nonlinear SVM on a dataset with only 500 K samples (Rahimi and Recht, 2007). To examine the quality of this dataset, we turn to four different types of deep neuron networks, two of which are ANNs while the others are SNNs. The structure of ResNet-18 and ResNet-34 (He et al., 2016) are applied in the experiments. The results of these experiments provide a benchmark for this dataset. It is noted that all of the accuracy mentioned here is top-1 test accuracy.

For ANNs, the two dimension convolutional neural network (2D-CNN) (Krizhevsky et al., 2012) has become a common tool for image classification. In order to train 2D-CNN on the ES-dataset, a common approach is to accumulate the events into event frames according to the time dimension and then reconstruct the gray images (Wu et al., 2020) for training. Here we use the Edge-Integral algorithm described in Figure 6 for reconstruction. The network structures we use here are the same as those in the original paper (He et al., 2016).

Meanwhile, regarding the time dimension as the depth, this dataset can also be considered as a video dataset, so the classic video classification methods can also be utilized, like 3D-CNN (Ji et al., 2013; Hara et al., 2018). By introducing the convolution of depth dimension, 3D-CNN has the ability of processing time-domain information. The structures we used are 3D-ResNet-18 and 3D-ResNet-34, and the convolution kernel is chosen to be 3 × 3 × 3, which ensures that the largest receptive field of the network can cover the whole time (depth) dimension.

**Figure 8:**
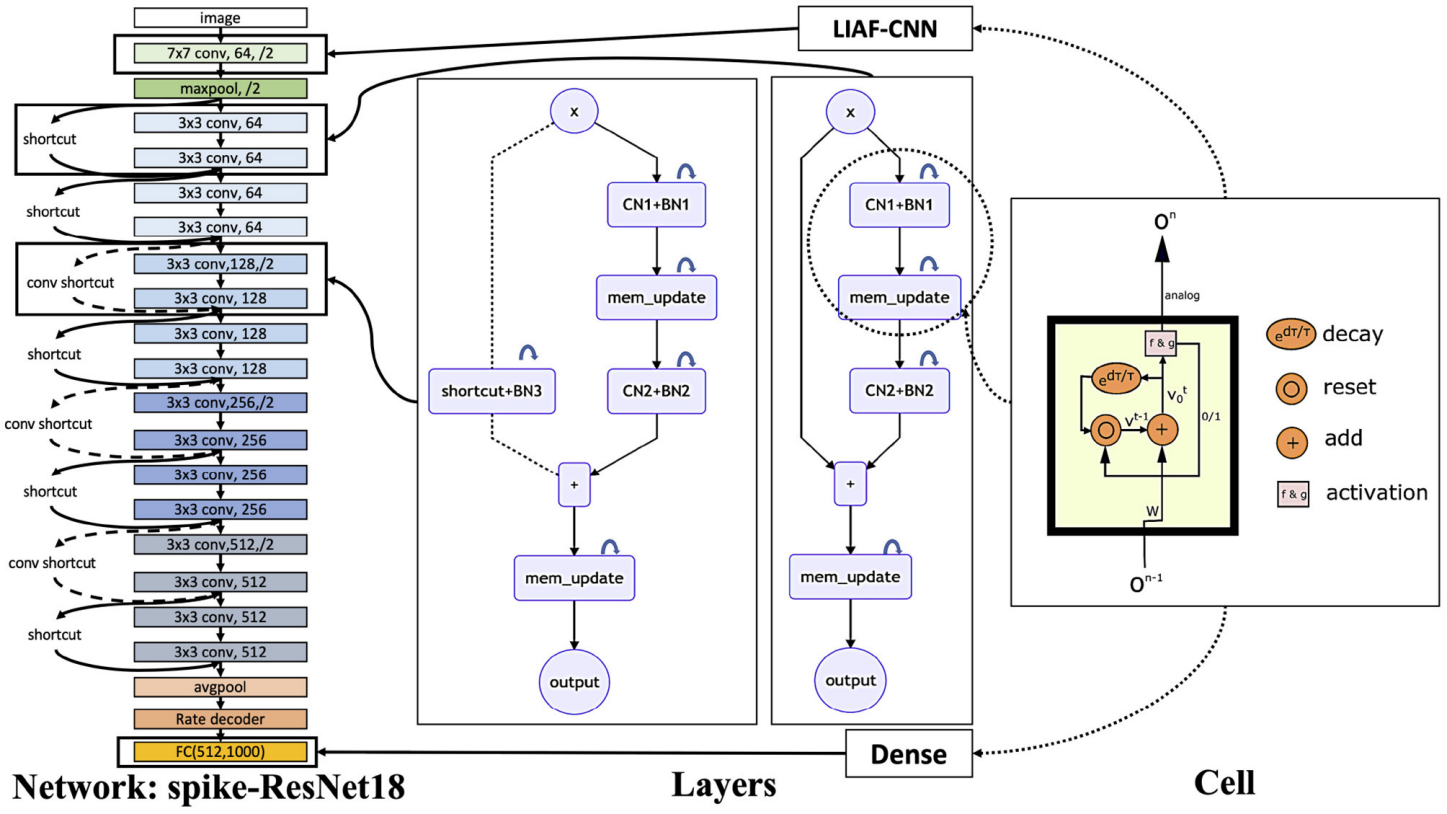
SNN structure used in the experiments. On the right, we show the internal structure of LIAF neurons. By changing synaptic connections, we can obtain a variety of layer structures, where CN denotes the convolutional layer and BN denotes the 3D-BatchNorm layer. Using these layers, we can build a scalable LIAF residual network structure.

For SNNs, we choose an SNN based on leaky integrate-and-fire (LIF) neurons (Dayan and Abbott, 2001) and an SNN based on leaky integrate-and-analog-fire (LIAF) (Wu et al., 2020) neurons. Rate coding (Adrian and Zotterman, 1926) is used to decode the event information because the significance of the specific time when the spikes appear in this dataset is weaker than the number of spikes. Both of the SNN models are trained using the STBP method (Wu et al., 2019) and sync-batch normalization (Ioffe and Szegedy, 2015), and the network structures similar to ResNet-18 and ResNet-34 are built as shown in Figure 8. The basic LIF (Dayan and Abbott, 2001) model is described in Equation (6),


(6) $$U=\{\begin{array}{ll} \tau_{m}\frac{dU}{dt}=-U+E_{L}+R_{m}I_{e} & if\;U<U_{thresh}\\ U_{reset} & U\geq U_{thresh} \end{array}$$

where U is the membrane potential, *E*_*L*_ is adjusted to make the resting potential match that of the cell *L* being modeled. *I*_*e*_ is the input current and the *R*_*m*_ is the membrane resistance. *U*_*reset*_ is a parameter adjusted according to the experiment data, and τ_*m*_ is the membrane time coefficient. The LIF neuron will fire a spike when *U* reaches the *U*_*thresh*_, and the spike can be {0,1} in LIF or an analog value in LIAF. Solving the model, we have the *U*(*t*), as shown in Equation (7).


(7) $$U(t)=E_{L}+R_{m}I_{e}+(U(0)-E_{L}-R_{m}I_{e})e^{-t/\tau_{m}}.$$

This equation does not take the reset action into consideration. For large-scale computer simulation, simplification is needed on this model and using the discrete LIAF/LIF model. Using *l* to present the layer index and *t* for the time, the LIAF model can be described by the following equations


(8) $$u_{0}^{t,l}=u^{t-1,l}+h(o^{t,l-1})+b^{l}$$

(9) $$s^{t,l}=f(u_{0}^{t,l})$$

(10) $$o^{t,l}=g(u_{0}^{t,l})$$

(11) $$u^{t,l}=u_{0}^{t_{k},l}d(s^{t,l})$$

where *h* is the weighted sum function of the input vector *o*^*t,l*−1^, which is related to the specific connection mode of synapses and is equivalent to *R*_*m*_*I*_*e*_. *s* is the spike used to reset the membrane potential to *U*_*reset*_ and *o*^*t, l*^ is the output of neurons to the next layer. We often use *d*(*x*) = τ(1 − *x*) for simplification in this model, where *d*(*x*) describes the leaky processing and τ is a constant relative to τ_*m*_. *f* is usually a threshold-related spike function, while *g* is selected to be a commonly used continuous activation function. If *g* is chosen as the same function as *f*, then the above model is simplified to the LIF model as


(12) $$u_{0}^{t,l}=u^{t-1,l}+h(o^{t,l-1})+b^{l}$$

(13) $$o^{t,l}=s^{t,l}=f(u_{0}^{t,l})$$

(14) $$u^{t,l}=u_{0}^{t_{k},l}\tau (1-s^{t,l})$$

To build a Spiking-ResNet model, we proposed the spiking convolutional layer and spiking-ResBlock structure. Only *h* in Equation (8) and Equation (12) needs to be changed to become different types of SNN layers. For the full-connection layer (or Dense), we choose $h\left(o^{t,l-1}\right)=W_{l}*o^{t,l-1}$, where *W*_*l*_ is the weight matrix of the *l* layer. In the convolutional layer, $h\left(o^{t,l-1}\right)=W_{l}⊗o^{t,l-1}$, where ⊗ is the convolution operation.

The residual block structure we used in the SNN is a little bit different. For better performance in deep SNN training, we add a 3D-BatchNorm layer on the membrane potential, where we treat the temporal dimension in the SNN as the depth of the general 3D data. In Figure 8, CN denotes the convolutional layer and BN denotes the 3D-BatchNorm layer, the *mem_update* layers are described by Equations (9)–(11) in LIAF-ResNet, and Equations (13)–(14) in LIF-ResNet. To keep the coding consistent, before each output of residual block, we add a *mem_update* layer.

The best test results are obtained based on the same set of hyper-parameters and different random seeds, which are shown in Table 3, and the results are listed in Table 4. During the training, the initial learning rate is 0.03, the optimizer is ADAM (Kingma and Ba, 2014), and the learning rate is optimized by the StepLR learning schedule. NVIDIA-RTX2080Tis are used for training and the Pytorch (Paszke et al., 2019) deep-learning framework is used for programming for all of these experiments.

**Table 3:** Hyper-parameter setting.

	**Names**	**Value**
Network	T	8
	Thresh	0.5
	Decay	0.5
Optimizer (ADAM)	Lr	3e-2
	β_1_, β_2_, λ	0.9,0.999,1e-8
Activation	Lens	0.5
StepLR	Nepoch	10
	α	0.2
Others	BatchSize	224^a^/160^b^
	Max Epoch	50

a *Used for the training of ResNet-18*.

b *Used for the training of ResNet-34*.

**Table 4:** Test results & benchmarks.

**Structure**	**Type**	**Test Acc/%**	**# of Para**
ResNet18	2D-CNN	41.030	11.68M
	3D-CNN	**43.140**	28.56M
	LIF (baseline)	39.894	11.69M
	LIAF	42.544	11.69M
ResNet34	2D-CNN	42.736	21.79M
	3D-CNN	45.380	48.22M
	LIF (baseline)	43.424	21.80M
	LIAF	**47.466**	21.80M

### Test Results

**Figure 9:**
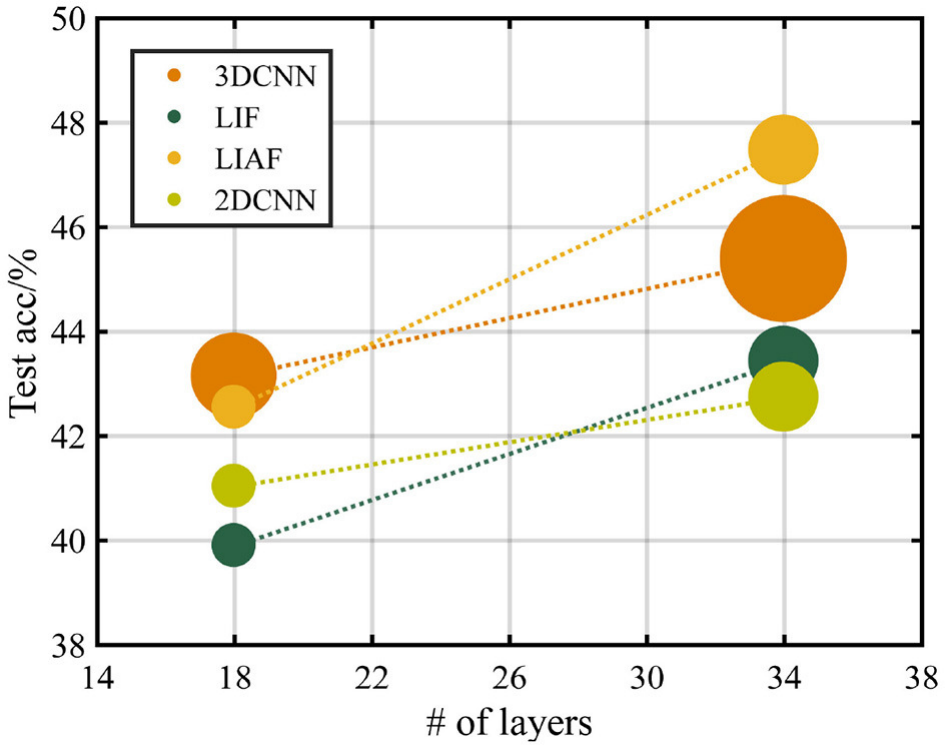
Testing accuracy with the structure of ResNet-18 and ResNet-34. The radius of the data points represents the relative size of the parameters of the networks.

As Table 4 shows, the highest test accuracy based on the ResNet-18 structure is obtained by the 3D-CNN, which is 43.140%. And the best result on ResNet-34 reaches 47.466% obtained by the LIAF-SNN. In order to show the relationship between parameter quantity and accuracy more intuitively, we provide Figure 9 and use the area of the disk to show the number of parameter, highlighting the efficiency of SNN. The experimental results of LIF-SNN, which is the traditional SNN model, will provide a baseline for this dataset, and we expect more advanced and large-scale SNNs or other neuromorphic algorithms to be tested on this dataset.

## Discussion

### Performance

Observing the results, we will find that the SNN models can obtain a relatively high classification accuracy with fewer parameters. The sparsity of the data in *ES-ImageNet* may lead to this phenomenon, for SNN can deal with spatiotemporal information efficiently, and a large number of parameters in an ANNs-based video classification algorithm (like 3D-CNN tested in this article) may cause over-fitting on this dataset.

We find the other two reasons for the accuracy loss. Wrongly labeled samples may also seriously interfere with the training progress. This problem is obvious in *ImageNet* (Northcutt et al., 2019), and we also found this problem when we conducted a manual inspection, but there is currently no good method for efficient and accurate screening. Another problem is the information loss. Given this problem, we propose several possible ways to optimize it. One is to filter out more samples with the highest and lowest information entropy (representing the largest noise rate and the smallest amount of information, respectively) in the training set. The other is to increase the number of time steps of the transformation, but it will increase the storage cost.

It should be noted that in the experiments we do not use any data augmentation method. In fact, placing event frames in random order, using dynamic time length, or processing each frame with random clipping are acceptable on this dataset and may bring significant performance boost. Research is under way on such data augmentation and pre-training technologies, and we hope more related research can use this dataset.

### Computation Cost

To make a more objective comparison, we also count and compare the theoretically minimum number of FP32 operands required by the feed-forward process of these networks by measuring the power consumption in a field-programmable gate array (FPGA).

Here we compare the number of necessary calculation operands required by the feed-forward process of eight different networks used in the main article. It should be noted that we calculate the number of floating-point multiplication operands and floating-point addition operands separately (not MACs), and the operands of normalization layers are not included in the calculation.

**2D-CNNs** use the ResNet structures with 18/34 layers, and most of the operands are bolstered by convolution layers. In this work, we compress and reconstruct the 4-dimensional event data in *ES-ImageNet* into 2-dimensional gray images, then feed them into 2D-CNNs. The process is then the same as the way we train a ResNet on *ImageNet*. In the network, the dimensions of the features change in the following order: [1(*channel*) × 224(*width*) × 224(*height*)] → (*maxpooling*)[64 × 110 × 110] → [64 × 55 × 55] → [128 × 28 × 28] → [256 × 14 × 14] → [512 × 7 × 7] → [512] → [1000].

**3D-CNNs** consider the depth dimension (Ji et al., 2013), and treat this dataset as a video dataset (Hara et al., 2018), so the feature is kept in four dimensions in ResBlocks. In the network, the dimensions of the features change in the following order: [2(*channel*) × 8(*depth*) × 224(*width*) × 224(*height*)] → (*maxpooling*)[64 × 8 × 110 × 110] → [64 × 4 × 55 × 55] → [128 × 2 × 28 × 28] → [256 × 1 × 14 × 14] → [512 × 1 × 7 × 7] → [512] → [1000].

The training procedure of **LIF-SNNs** is like running eight 2D-CNNs along with the processing of the last moment of membrane potential memory information and the spikes inputs for every layer, then averaging the spike trains in the time dimension in the final linear layer to decode the spiking rate. These networks keep the data in four dimensions with *T* = 8 unchanged until the decoding layer, so the dimensions of the features change in the following order: [2(*channel*) × 8(*T*) × 224(*width*) × 224(*height*)] → (*maxpooling*)[64 × 8 × 110 × 110] → [64 × 8 × 55 × 55] → [128 × 8 × 28 × 28] → [256 × 8 × 14 × 14] → [512 × 8 × 7 × 7] → [8(*depth*) × 512](*ratedecoded*) → [512] → [1000].

The training procedure of **LIAF-SNNs** is almost the same as **LIF-SNNs**, the only difference with **LIF-SNNs** is that they do not use binary spikes to convey information between layers, instead they use an analog spike. The dimensions of the features are the same as the ones in **LIF-SNNs**.

It is worth noting that since the input of **LIF-SNNs** is only 0 and 1, convolution does not need to compute floating-point multiplication, but does need to compute addition under a limited combination. As a large number of zeros appear in the input of each layer of **LIF-SNNs**, the optimization of sparse input for **LIF-SNNs** has become a formula in SNN accelerators. Therefore, in order to make a fair comparison, we can use the average fire rate obtained in the experiment multiplied by the input of the SNN as the proportion of the number of floating-point numbers that need to participate in the addition calculation (FP32 +), so as to estimate the actual amount of computation of SNNs and CNNs. We observe that the fire rate always shows a downward trend with the increase of training epochs, which means a decrease of meaningless spikes.

**Figure 10:**
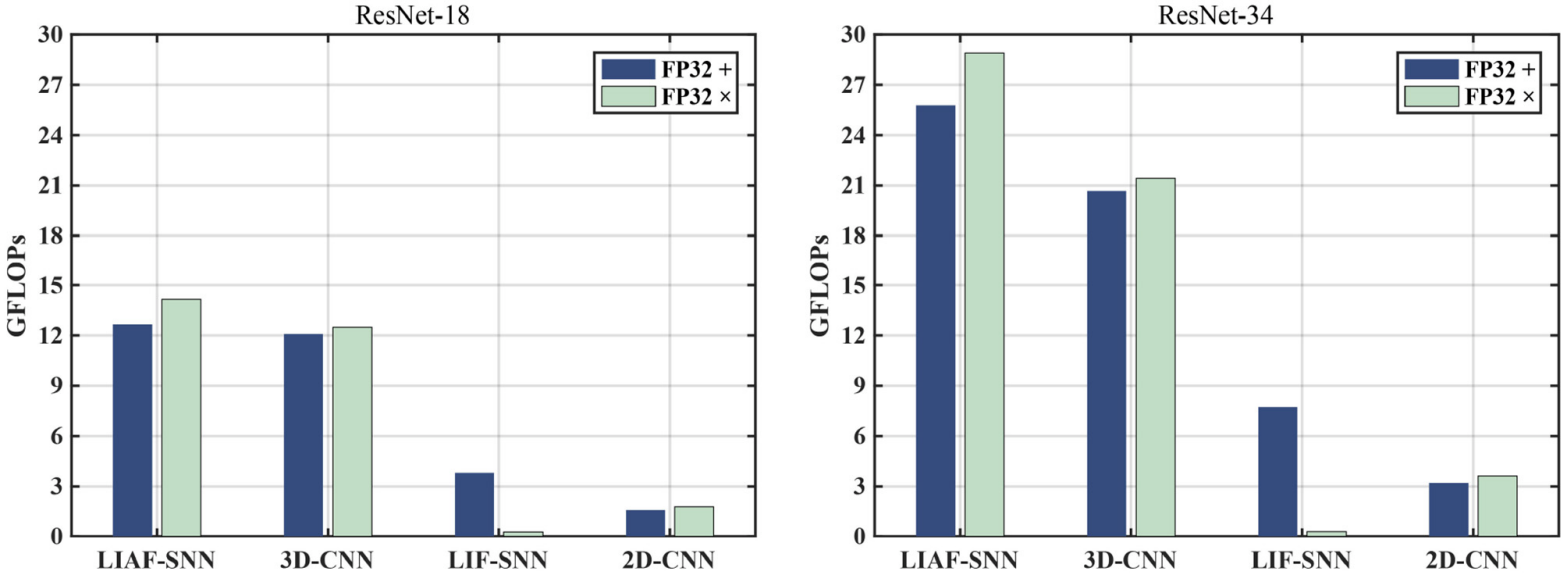
The comparison of FP32 addition (FP32 +) and FP32 multiplication (FP32 x) operations in the feed-forward process between the models we use in the experiments. It should be noted that the number of FP32+ operands of LIF have been multiplied by a sparsity factor (30%) for a fair comparison.

In this experiment, the initial fire rate of SNN is no larger than 30%, and with the increase of training epochs, the fire rate would gradually reduce to less than 10%. To compare the results of SNN in the worst case, we take 30% as the sparsity rate. Based on these conditions we can get Figure 10.

**Figure 11:**
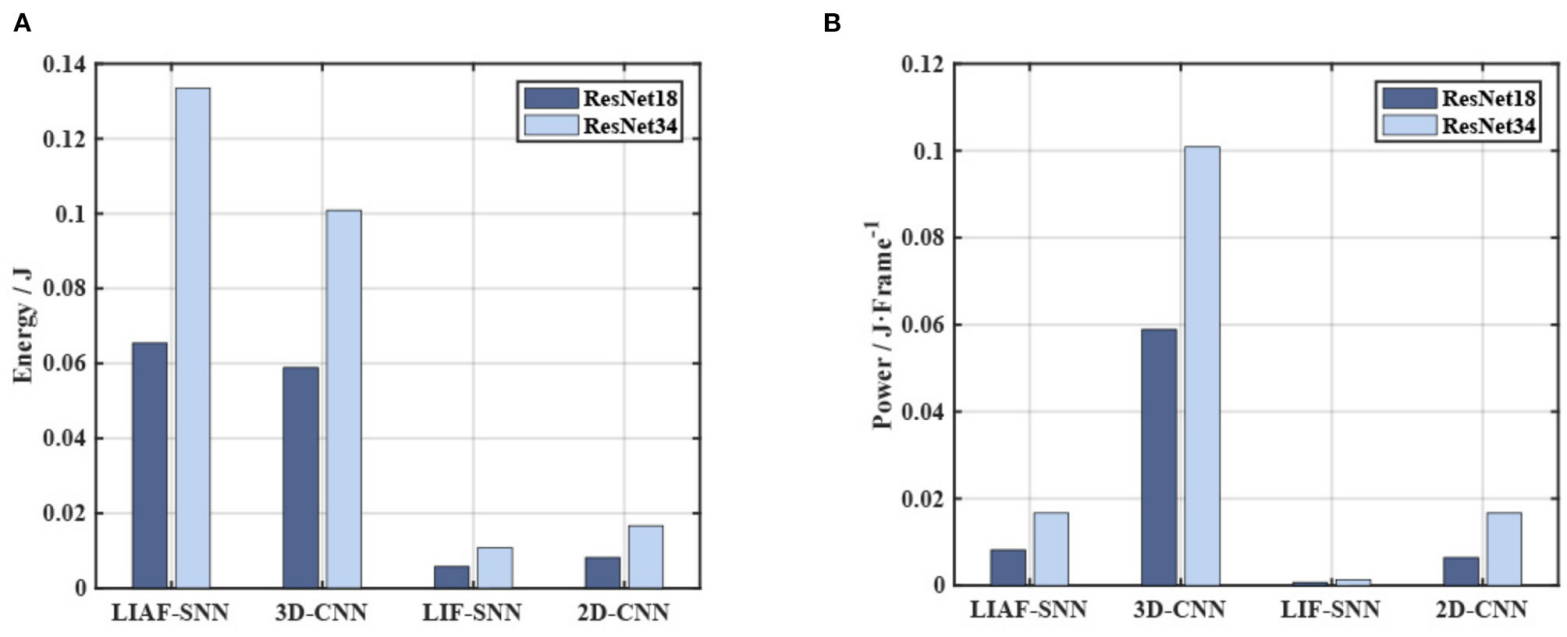
Energy and power consumption in the experiments. **(A)** The comparison on energy cost. The LIF-SNNs have shown a significant advantage in energy consumption, whose energy cost is half of that of the CNN. **(B)** The power of each model in the *Frame* time unit is also the energy required for one feed-forward process.

One of the advantages of an SNN compared with an ANN is its power consumption (especially in SNNs' accelerators). On FPGAs the SNNs could have a significant power advantage if the training algorithm is well designed. The data in Wu et al. (2019) about the basic operands' power consumption can provide an estimation of the power consumption of the networks in the experiments. Each FP32 + operation requires 1.273 pJ of energy, and each FP32 operation requires 3.483 pJ of energy. Then we can get the result in Figure 11A. In addition, we also give the power comparison commonly used for SNNs (Deng et al., 2020) in Figure 11B, where we calculate the energy for each feed-forward process (so we call it power). It should be noted that SNNs need *T* frames to give one prediction, and both the 3D-CNN and 2D-CNN give one prediction based on one frame.

These results also support the SNNs' energy advantages in this task. For these reasons, we think *ES-ImageNet* would be an SNN-friendly dataset. We still hope that more ANNs algorithms will be proposed to solve these challenges elegantly and efficiently, which may also provide guidance for the development of SNNs.

### Limitations

For the conversion algorithm, we generate temporal features by applying artificial motion to static images like most conversion methods, which is still different from the real scene. It is the limitation for those dynamic datasets derived from static data. In addition, in order to compress the volume of the dataset and extract more information, we reduce the randomness of data during generation, thus losing a certain feature of DVS camera recording but being more friendly to SNNs.

In the analysis part, due to the limitation of mathematical tools, the 2D-Entropy we adopt can reflect only the amount of information, not the amount of effective information. Therefore, it can only be used as a reference rather than a standard. In addition, the reconstruction method and the compression method used in measurement would influence the information, though we have compared them as fairly as possible.

In the training method, due to the limitation of hardware conditions and algorithms, we can only provide the benchmarks of SNNs and ANNs based on ResNet-18 and ResNet-34. It is hoped that more research will participate in the training of larger and better models.

## Conclusion

In this paper, we provide a new neuromorphic vision dataset named *ES-ImageNet* for event-based image classification and validation of SNN algorithms. We proposed a method called ODG, transforming a famous image classification dataset *ILSVRC2012* into its event-based version with a method called Edge-Integral to reconstruct the corresponding gray images based on these event streams. The ODG method includes a carefully designed image movement, which results in the value changes in the HSV color space and provides spatial gradient information. This algorithm can efficiently extract the spatial features to generate event streams.

For testing the properties of datasets, we use the Edge-Integral method to exhibit some of the reconstructed samples, and also calculate the 2D-Entropy distribution of the dataset. Furthermore, a comparative experiment is conducted using 2D-CNN, 3D-CNN, LIF-SNN, and LIAF-SNN, these results provide a benchmark for later research, and also confirm that this dataset is a suitable validation tool for SNNs.

This dataset solves the problem of lacking a suitable large-scale classification dataset in the SNNs' research field. It fills in this gap of a suitable dataset for the verification of large-scale SNNs so that the corresponding algorithm is expected to be better optimized, and more SNNs' structures and training algorithms will be explored, thereby promoting practical applications of SNNs.

## Data Availability Statement

The datasets ES-ImageNet (100GB) for this study can be downloaded at: https://cloud.tsinghua.edu.cn/d/94873ab4ec2a4eb497b3. The converted event-frame version (40GB) can be found at: https://cloud.tsinghua.edu.cn/d/ee07f304fb3a498d9f0f/. The codes can be found at: https://github.com/lyh983012/ES-imagenet-master.

## Author Contributions

YL and GL conceptualized the work. YL designed the research. YL, WD, and SQ designed and conducted the experiment. YL analyzed data. LD and GL supervised the work. All authors wrote the manuscript.

## Funding

This work is partially supported by the National Key R&D Program of China (2020AAA0105200, 2018AAA0102604, and 2018YFE0200200), the National Science Foundation of China (61876215), the Beijing Academy of Artificial Intelligence (BAAI), a grant from the Institute for Guo Qiang, Tsinghua University, and Beijing Science and Technology Program (Z191100007519009), the open project of Zhejiang laboratory, and the Science and Technology Major Project of Guangzhou (202007030006).

## Conflict of Interest

The authors declare that the research was conducted in the absence of any commercial or financial relationships that could be construed as a potential conflict of interest.

## Publisher's Note

All claims expressed in this article are solely those of the authors and do not necessarily represent those of their affiliated organizations, or those of the publisher, the editors and the reviewers. Any product that may be evaluated in this article, or claim that may be made by its manufacturer, is not guaranteed or endorsed by the publisher.
